# Pain drawings predict outcome of surgical treatment for degenerative disc disease in the cervical spine

**DOI:** 10.1080/03009734.2017.1340372

**Published:** 2017-07-18

**Authors:** Anna MacDowall, Yohan Robinson, Martin Skeppholm, Claes Olerud

**Affiliations:** a Department of Surgical Sciences, Uppsala University Hospital, Uppsala, Sweden;; b Department for Learning, Informatics, Management and Ethics, Karolinska Institutet, Stockholm, Sweden

**Keywords:** Cervical spine, Neck Disability Index, outcome, pain drawing, repeatability, surgical treatment

## Abstract

**Introduction:**

Pain drawings have been frequently used in the preoperative evaluation of spine patients. For lumbar conditions comprehensive research has established both the reliability and predictive value, but for the cervical spine most of this knowledge is lacking. The aims of this study were to validate pain drawings for the cervical spine, and to investigate the predictive value for treatment outcome of four different evaluation methods.

**Methods:**

We carried out a *post hoc* analysis of a randomized controlled trial, comparing cervical disc replacement to fusion for radiculopathy related to degenerative disc disease. A pain drawing together with Neck Disability Index (NDI) was completed preoperatively, after 2 and 5 years. The inter- and intraobserver reliability of four evaluation methods was tested using κ statistics, and its predictive value investigated by correlation to change in NDI.

**Results:**

Included were 151 patients, mean age of 47 years, female/male: 78/73. The interobserver reliability was fair for the modified Ransford and Udén methods, good for the Gatchel method, and very good for the modified Ohnmeiss method. Markings in the shoulder and upper arm region on the pain drawing were positive predictors of outcome after 2 years of follow-up, and markings in the upper arm region remained a positive predictor of outcome even after 5 years of follow-up.

**Conclusions:**

Pain drawings were a reliable tool to interpret patients’ pain prior to cervical spine surgery and were also to some extent predictive for treatment outcome.

## Introduction

Pain drawings have been a common tool allowing patients to communicate pain without the necessity of an elaborate language for quite some time. In low-back-pain patients, pain drawings have been analysed qualitatively into organic or non-organic drawings by penalty point systems or general impression (1–4). Several quantitative analyses have also investigated how widespread or localized the pain markings on the pain drawing are (5–7). Until now most investigations focused on low-back-pain patients even though pain drawings are frequently used in neck pain patients as well. Gioia et al. (8) found stronger levels of agreement between pain drawings and degenerative changes on MRI of the cervical spine compared to the lumbar spine.

Cervical radiculopathy is caused by degenerative changes such as disc herniation or foraminal narrowing due to decreased disc height, pleated ligament, and osteophyte formation in the uncovertebral- and or facet joints. The most commonly affected nerve root is the C7, secondly the C6. The symptoms are neck pain with arm pain in the same distribution area as the affected nerve (9). To our knowledge a thorough study about the role of pain drawings in preoperative assessment for cervical degenerative disc disease (DDD) has not been done. In a recent study about pain drawings in patients with cervical radiculopathy we concluded that the pain drawing was affected by both pain intensity and anxiety/depression (10).

This study was designed to validate pain drawings of neck pain patients in terms of inter- and intraobserver reliability of four different interpretation scores and to evaluate whether these interpretation scores are predictive for treatment outcome.

### Patients and methods

This study was a *post hoc* analysis of a prospective randomized controlled trial (RCT) of 151 patients from three hospitals in Sweden during 2007 through 2010. The patients suffered from radiculopathy due to DDD and were randomized after exposure and decompression to either artificial disc replacement (ADR) (Discover^TM^, DePuy Spine, Johnson & Johnson) or plated fusion using autologous iliac crest graft. Inclusion and exclusion criteria as well as 2-year results have been published previously (11). On the day before surgery the patients completed a questionnaire with demographic details, a pain drawing, and the Neck Disability Index (NDI) (Table 1).

**Table 1.** Demographics at baseline.

Patient characteristics	Total *n*	
Age, median (min, max)	130	46 (31, 61)
Women/men, *n*	130	67/63
Smokers, *n* (%)	130	39 (30)
Non-smokers, *n* (%)	130	91 (70)
In work, *n* (%)	128	111 (87)
Not in work, *n* (%)	128	17 (13)
ΔNDI 2y, median (min, max)	122	–22 (–66, 22)
ΔNDI 5y, median (min, max)	115	–28 (–74, 28)

NDI: Neck Disability Index; ΔNDI 2y: NDI at 2 years of follow-up minus preoperative NDI; ΔNDI 5y: NDI at 5 years of follow-up minus preoperative NDI.

The study was approved by the Regional Ethical Review Committee in Stockholm (Dnr: 2006/1266-31/3). Patient informed consent was obtained before randomization. The study was registered at ISRCTN (registration number: 44347115).

#### Data collection

The pain drawing developed by Spangfort (12), which is a modified version from Ransford et al. (1), was used. The test consists of a front and back outline drawing of the human body. Patients indicate the distribution and the character of their present pain using six different symbols: dull, burning, numbness, stabbing or cutting, pins and needles, and cramping (Appendix 1). The test was completed on the day before surgery, but the result of the test was not revealed to the surgeon at the time of the operation.

Three spine surgeons—one with <5 years of experience, one with 10 years of experience, and one with >30 years of experience—scored the pain drawings independently to determine the interobserver reliability. The less experienced observer performed a second scoring 1 month after the first scoring, blinded from the previous results of the first scoring, to determine intraobserver reliability. For evaluation of the pain drawings we used the penalty point system by Ransford (1), the visual inspection method by Udén (2), the grid assessment method by Gatchel (7), and scoring into body surfaces by Ohnmeiss (5). To measure treatment outcome we used NDI (13,14).

*Penalty point system by Ransford*. The pain drawing was assigned points for the following characteristics: unreal drawings (indications of pain in patterns inconsistent with radicular symptoms), drawings showing ‘expansion’ or ‘magnification’ of pain (indicating pain outside the drawing of the body), ‘I particularly hurt here’ indicators (using arrows or extra words to emphasize pain intensity), ‘Look how bad I am’ indicators (a tendency to demonstrate total body pain). A score of two points or less was regarded as normal (Appendix 2). The penalty point system by Ransford was modified to the cervical spine and is henceforth nominated the modified Ransford method.

*Visual inspection method by Udén*. The visual inspection method by Udén was modified to the cervical spine as follows:

- Neurogenic (N)—the pain drawing shows pain in the arm and/or shoulder as in typical nerve root pain.
- Possible neurogenic (PN)—the pain drawing shows some aberrations from a classic nerve root syndrome.
- Non-neurogenic (NN)—the pain has a distribution that could not be explained by radiculopathy.
- Possible non-neurogenic (PNN)—the pain drawing shows very little resemblance with a nerve root pain and is therefore hard to categorize into the other groups above.
- The visual inspection method by Udén is henceforth nominated the modified Udén method.

*Grid assessment method by Gatchel*. The pain drawing was divided with bilaterally symmetrical grids with small boxes of approximately equal area. The grid over the human figure was copied onto a transparent plastic template and placed over each completed pain drawing for scoring. The number of boxes filled in by markings was counted.

*Scoring into body surfaces by Ohnmeiss*. The method was modified for cervical use, hence the pain drawing was divided into the following five regions: neck, head, upper trunk (scapula region), upper arm, and lower arm. Markings on the elbow or wrist non-contiguous with neck or arm pain were disregarded because they may indicate joint problems (5). We used a transparent plastic template with the human figure containing the boundaries to place over each completed pain drawing for scoring (Appendix 1). The scoring into body surfaces by Ohnmeiss is henceforth nominated the modified Ohnmeiss method.

*Neck Disability Index*. The NDI is a self-administered questionnaire with 10 items measuring disability in patients with neck pain. The questions cover daily activities such as ability to dress, lift heavy objects, read, work, drive a car, sleep, and perform leisure activities as well as investigating the amount of pain, headache, and concentration abilities. Each item is scored from 0 to 5. The disability is more severe with higher scores. Maximum score is 50 (13). The number score can also be transformed to percentage score, which means that the range is 0% to 100%. The NDI has been validated, and the minimum clinically important difference is 7.5–8.5 (13,15) or 17.3% (16). The NDI was administered to the patients the day before surgery and again at the 2-year and 5-year follow-up.

#### Statistical methods

The modified Ransford and Udén methods were dichotomized to neurogenic/non-neurogenic according to the original articles. The Gatchel method was dichotomized according to Takata et al. (Table 2) (17).

**Table 2.** Principles of dichotomization of the various methods.

Method	Neurogenic	Non-neurogenic
Ransford (penalty points)	0–2	3+
Udén	N, PN	NN, PNN
Gatchel (ticked boxes, *n*)	0–19	20+

*Reliability*. For each dichotomous method, reliability was assessed in the following ways: The percentage total agreement between the three observers was computed. This is simply the per cent of the patients for which all three observers gave the same result. Light’s κ (kappa) (18) was computed for the same three observers as above. This is defined as the average of all three possible pairwise Cohen’s κ. Cohen’s κ (19) was computed for two pairs of observers: very experienced versus less experienced; same observer (less experienced) at two different occasions.

κ ≤ 0.20 is considered to be poor agreement, κ 0.21–0.40 is fair agreement, κ 0.41–0.60 is moderate agreement, κ 0.61–0.80 is good agreement, and κ 0.81–1.00 equals very good agreement.

*Validation*. The following values were correlated to the pain drawings: preoperative NDI and absolute change in NDI (ΔNDI, i.e. 2-year NDI minus preoperative NDI, and 5-year NDI minus preoperative NDI).

The inferential part was done only for the dichotomous/dichotomized methods. For each such method and observer (including a ‘total’ one, pooling the results from all three observers), the endpoint values for the N and NN groups were compared. The endpoint mean for the N group was subtracted from the endpoint mean in the NN group. For the modified Ohnmeiss method, the comparison was instead between groups 0 (no pain markings) and 1 (with pain markings) separately for each body surface region. The endpoint mean for the group 0 was subtracted from the endpoint mean in group 1. Positive values correspond to larger values for the NN group or, for the modified Ohnmeiss method, for group 1. For the endpoints representing a change (ΔNDI) this typically means less negative values, i.e. closer to zero, suggesting that the NN group (or group 1) performed ‘worse’. That is because the patients that improve after surgery get a negative ΔNDI, i.e. the preoperative NDI value is high and is subtracted from the postoperative NDI value that is low. The more negative ΔNDI value, the greater is the patient’s improvement.

The target parameter was the difference in means. Confidence intervals (CI) and *P* values were computed using bootstrap with *B* = 10,000 bootstrap replicates and the percentile method (20). *P* values of <0.05 were considered significant. For the ‘total’ observer, we resampled patients (i.e. triplets of values) rather than individual values, reflecting the dependence between values for the same patient.

Missing data were handled using ‘available cases’. Hence only patients without missing values for the variables used in the analysis at hand were included. Consequently, the populations the various analyses were based on are not the same.

No correction was done for multiple testing/estimation.

All statistical analyses were performed in R (21), version 3.1.0 (2014-04-10), x86_64-w64.

## Results

Of the 151 patients included in the RCT, 20 patients had missing data for pain drawings. One pain drawing was incorrectly given after the operation. Three patients were lacking preoperative NDI, five were lacking 2-year NDI, and 12 were lacking 5-year NDI.

The distributions of N and NN pain drawings were equal for the modified Ransford and Gatchel methods, with ∼50% of the patients in each group compared to the modified Udén method (N, 72%; NN, 28%). There was an uneven distribution in the modified Ohnmeiss regions, where 80% had marked pain in the neck, 95% in the shoulder, 91% in the upper arm, and 97% in the lower arm (Table 3).

**Table 3.** Percentage pain drawings assessed to each group.

Method	Percentage of assessedpain drawings
Ransford	
Neurogenic	49.6%
Non-neurogenic	50.4%
Udén	
Neurogenic	72.1%
Non-neurogenic	27.9%
Gatchel	
Neurogenic	44.6%
Non-neurogenic	55.4%
Ohnmeiss	
Head	
0	78.3%
1	21.7%
Neck	
0	20.0%
1	80.0%
Shoulder	
0	4.6%
1	95.4%
Upper arm	
0	9.2%
1	90.8%
Lower arm	
0	3.3%
1	96.7%
Bilateral	
0	75.6%
1	24.4%

The agreement between all three observers was fair in the modified Ransford and Udén methods (κ, 0.29 and 0.36, respectively), good in the Gatchel method (κ, 0.79), and very good in the modified Ohnmeiss method (κ, 0.8 to 1.0). The re-evaluation by the same observer was good in the modified Ransford method (κ, 0.72), moderate in the modified Udén method (κ, 0.50), and very good in the Gatchel and modified Ohnmeiss methods (κ, 0.87 to 1.00) (Table 4).

**Table 4.** Results of the reliability analysis.

Method	% agreement(all three evaluators)	Light’s κ(all three evaluators)	Cohen’s κ(less very experienced evaluator)	Cohen’s κ(re-evaluation same observer)
Ransford	42	0.29	0.50	0.72
Udén	61	0.36	0.43	0.50
Gatchel	85	0.79	0.80	0.88
Ohnmeiss				
Head	90	0.80	0.89	0.87
Neck	91	0.81	0.78	0.91
Shoulder	98	0.88	0.90	0.82
Upper arm	95	0.81	0.89	0.95
Lower arm	100	1.00	1.00	1.00
Bilateral	93	0.88	0.86	0.98

Preoperative NDI was higher in the NN groups compared to the N groups in the modified Ransford (mean, 5.4; 95% CI, 2.0 to 8.7), Udén (mean, 4.2; 95% CI, 0.5 to 8.0), and Gatchel methods (mean, 6.9; 95% CI, 2.4 to 11.5). In the modified Ohnmeiss groups, the preoperative NDI was higher among the patients who had marked pain in the head region compared to those who did not (mean, 7.5; 95% CI, 2.8 to 12.2) (Table 5).

**Table 5.** Results from method comparison.

Method	NDI, preopMean diff. (95% CI) *P*	ΔNDI 2 y (2 y NDI–preop NDI)Mean diff. (95% CI) *P*	ΔNDI 5 y (5 y NDI–preop NDI)Mean diff. (95% CI) *P*
Ransford	5.4 (2.0, 8.7) 0.0031	–0.8 (–5.0, 3.5) 0.72	0.7 (–4.4, 5.6) 0.78
Udén	4.2 (0.5, 8.0) 0.031	1.9 (–3.6, 7.2) 0.50	2.0 (–4.0, 7.7) 0.49
Gatchel	6.9 (2.4, 11.5) 0.002	2.0 (–1.6, 5.7) 0.55	3.6 (–3.1, 9.7) 0.27
Ohnmeiss			
Head	7.5 (2.8, 12.2) 0.002	–0.5 (–8.4, 7.8) 0.91	–1.5 (–10.3, 7.5) 0.74
Neck	5.3 (–1.5, 12.1) 0.12	4.1 (–4.1, 12.5) 0.32	4.5 (–4.1, 13.4) 0.31
Shoulder	11.4 (–4.1, 26.3) 0.13	–13.0 (–18.6, –5.9) < 0.001	–8.6 (–21.9, 9.3) 0.29
Upper arm	–0.2 (–8.5, 6.9) 0.96	–10.4 (–18.3, –2.0) 0.014	–12.1 (–17.4, –6.1) < 0.001
Lower arm	8.6 (–10.0, 23.8) 0.31	–2.1 (–11.8, 10.2) 0.72	1.7 (–16.4, 12.3) 0.80
Bilateral	4.1 (–1.2, 9.6) 0.13	2.1 (–5.9, 10.0) 0.60	3.8 (–4.7, 11.8) 0.37

The values are:

The mean difference between preoperative NDI in group N and NN, group 1 and 0.

The mean difference between ΔNDI in group N and group NN, group 1 and group 0.

The mean difference between ΔNDI at the 5-year follow-up, in group N and group NN, group 1 and group 0.

Difference in means presented for each method. The endpoint mean for the N group is subtracted from the endpoint mean in the NN group. For the Ohnmeiss method, the endpoint mean for the group 0 (no markings) was subtracted from the endpoint mean in group 1 (with markings). Hence positive values correspond to larger values on preoperative NDI for the NN group or (for Ohnmeiss) for group 1. For the endpoints representing a change (ΔNDI) this typically means less negative values, i.e. closer to zero, suggesting that the NN group (or group 1) performs ‘worse’.

The patients with markings in the shoulder region (mean, –13.0; 95% CI, –18.6 to –5.9) and upper arm region (mean, –10.4; 95% CI, –18.3 to –2.0) improved more from surgery at the 2-year follow-up than the patients with no markings in these regions. At the 5-year follow-up there were no remaining differences in improvement for those with markings in the shoulder region. For the patients with markings in the upper arm region the greater improvement was sustained even after 5 years (mean, –12.1; 95% CI, –17.4 to –6.1) (Table 5).

The preoperative NDI differences between the N and NN groups had no effect on the clinical outcome 2 and 5 years after surgery. ΔNDI improved above the minimum clinically important difference (MCID) of 17% for NDI (16), in both the N and NN groups of the modified Ransford, the modified Udén, and the Gatchel methods. In the modified Ohnmeiss method the ΔNDI of the patients without markings of pain in the shoulder and upper arm region did not improve above the MCID (Table 6).

**Table 6.** ΔNDI at 2 years and 5 years postoperative, for all evaluation methods and for the totality of all observers.

Method	ΔNDI 2 y median(range)	ΔNDI 5 y median(range)
Ransford		
Neurogenic	–22 (–66, 22)	–30 (–75, 28)
Non-neurogenic	–23 (–66, 22)	–28 (–74, 12)
Udén		
Neurogenic	–24 (–66, 22)	–30 (–72, 28)
Non-neurogenic	–18 (–66, 12)	–22 (–74, 8)
Gatchel		
Neurogenic	–22 (–66, 12)	–32 (–70, 28)
Non-neurogenic	–24 (–66, 22)	–28 (–74, 12)
Ohnmeiss		
Head		
0	–22 (–66, 12)	–28 (–74, 28)
1	–26 (–66, 22)	–30 (–72, 12)
Neck		
0	–24 (–66, 6)	–31 (–74, 4)
1	–22 (–66, 22)	–28 (–72, 28)
Shoulder		
0	–8 (–22, –4)	–16 (–58, 2)
1	–24 (–66, 22)	–30 (–74, 28)
Upper arm		
0	–16 (–42, 6)	–16 (–48, –2)
1	–26 (–66, 22)	–30 (–74, 28)
Lower arm		
0	–18 (–32, –12)	–36 (–40, –10)
1	–22 (–66, 22)	–28 (–74, 28)
Bilateral		
0	–24 (–66, 22)	–30 (–72, 28)
1	–18 (–66, 12)	–20 (–74, 4)

Entries are median (min, max).

## Discussion

This validation study documented for the first time the reliability of cervical pain drawings and the associations between cervical pain drawings and surgical treatment outcome. In our study the agreement between two observers of the modified Ransford and the Udén methods was moderate. This contradicts the validation of the original methods by Ransford and Udén on low-back pain patients. Von Baeyer et al. (22) presented 87% agreement (correlation coefficient, 0.97) with the Ransford method. Udén’s method has been validated as 71%–78% agreement (κ, 0.7–0.9) by several authors (2,23–26). The Gatchel and modified Ohnmeiss methods did not require subjective interpretation by the evaluator; consequently the interobserver reliability was very good.

Hayashi et al. (24) presented no association between ‘non-organic’ pain drawings in neck-pain patients and (non-surgical) treatment outcome. Within the same study Hayashi et al. associated non-organic pain drawings with poor treatment outcome in low-back-pain patients. Consequently, studies on pain drawings in low-back-pain patients are not applicable to neck-pain patients. In the present study on patients with cervical radiculopathy the non-neurogenic pain drawing groups had higher preoperative NDI, but they benefited from surgery equally as much as the neurogenic pain drawing groups.

It seems arbitrary if we can read something valuable out of the patients’ pain drawing or not. Mann et al. (27) let low-back-pain physicians diagnose patients into one out of five disorders (benign back pain, herniation of the nucleus pulposus, spinal stenosis, serious underlying disorders, and psychogenic regional pain disturbance) by interpreting the patients’ pain drawing. The accuracy was only 51%. In our study a patient group with the specific diagnosis of cervical radiculopathy was selected. More than 90% of the patients had made markings on the upper and lower arm. Patients who also marked pain for other potential diagnoses, such as knee osteoarthritis or lumbar spinal stenosis, would have their drawings classified as non-neurogenic pain drawings according to the modified Ransford method or the dichotomized version of the Gatchel method. Hence, the modified Ohnmeiss method only considered pain in a specific region, disregarding other potential pain-generating diagnoses; this method was the only one with clear correlations to surgical treatment outcome. While the modified Ohnmeiss method has also previously shown associations to psychological impairment (10), this is a reliable method that can be applied to assess cervical pain drawings.

To avoid bias by influencing each other, the three spine surgeons that scored the pain drawings in our study did not discuss the written directions beforehand. Such discussions, or a trial period on ‘dummy patients’ to coordinate the interpretations, could possibly have made the assessors more uniform, which may have improved the interobserver agreement. On the other hand, our scenario reflects more how the methods would be used in practice, how interpretations between different assessors would in fact be spread.

Markings in the arm region on the pain drawing have previously been well associated to the presence of herniated nucleus pulposis on MRI (κ, 0.6) (8). In our study on patients with cervical radiculopathy, pain markings in the lower arm region on the pain drawings did not associate with surgical treatment outcome. Due to clear inclusion criteria, one limitation with such a homogeneous study population was that 97% of the patients had marked pain in the lower arm region and only 3% had not. An uneven distribution was also seen in the shoulder region (96% with markings) and upper arm region (91% with markings). This reflected the level of nerve root compression that was between C5 and C7 in 98% of the patients. With bootstraps there was higher chance of getting a valid result despite uneven distribution (20), though we recognize this as a potential weakness of statistical evidence. Therefore, one should be cautious in generalizing these results to other diagnoses.

The statistical calculations were done without correction for multiple testing/estimation. We hereby accept the risk of making a type one error. Since we did not compute so many variables we estimated that with a correction for multiple testing the results would be too conservative, hence carrying the risk of making a type two error. According to false discovery rate, we had only approximately 1% risk of making a type one error, which we appraised to be a small risk (28).

Scoring pain drawings with the modified Ohnmeiss method had very good inter- and intraobserver reliability. This method was also valuable in predicting surgical treatment outcome in patients with nerve root compression in the cervical spine. Preoperative markings in the shoulder and upper arm region on the pain drawing had superior outcome 2 years after surgery compared to no pain markings in those regions. Preoperative markings in the upper arm region on the pain drawing remained a positive predictor for superior treatment outcome even 5 years after surgery.

## Appendix 2. Penalty point system by Ransford modified for the cervical spine.

**Hypothesis for Evaluating Pain Drawings in the Cervical Spine**

Patient nb: _____________ □ pre. op □ 3 m (4w) □ 2 y (1y)

A patient with poor psychometrics may show this by:

1.*Unreal drawings* (poor anatomic localization, scores 2 unless indicated; bilateral pain not weighed unless indicated)

- total arm pain
- lateral whole arm pain (tuberculum majus area and lateral upper arm allowed)
- circumferential upper arm pain
- bilateral anterior under arm pain (unilateral allowed)
- circumferential hand pain (scores 1)
- bilateral hand pain (scores 1)
- use of all four modalities suggested in instructions (we feel patient is unlikely to have ”burning areas,” stabbing pain, pins and needles, and numbness all togetherSCORE: ______

2.*Drawings showing* ”expansion” or ”magnification” of pain (may also represent unrelated symptomatology; bilateral pain not weighted)

- neck pain radiating to head, shoulder, thoracic or lumbar spine (each scores 1; scapula pain allowed)
- elbow pain
- wrist pain
- pain drawn outside the outline; this is particularly good indication magnification (scores 1 or 2 depending on extent)SCORE: ______

3.”*I Particularly Hurt Here*” indicators

Some patients needing to make sure the physician is fully aware of the extend of symptoms may: (each category scores 1; multiple use of each category is not weighted)

- add explanatory notes
- circle painful areas
- draw lines to demarcate painful areas
- use arrows
- go to excessive trouble and detail in demostrating the pain areas (using the symbols suggested)SCORE: ______

4.”*Look How Bad I Am*” indicators

Additional painful areas in the trunk, head, lumbar spine or lower extremities drawn in. Tendency towards total body pain (scores 1 if limited to small areas, otherwise scores 2).

SCORE: ______

TOTAL: _____  □ normal (score > 2)  □ suggests poor psychometrics
